# The anterior reach of the modified Star Excursion Balance Test shows a strong relationship with ankle dorsiflexion in young athletes: a cross-sectional observational study

**DOI:** 10.1186/s40001-025-03625-w

**Published:** 2025-12-05

**Authors:** Raphael Schmidt de Mesquita, Marina Leandro Machado, Vitor Guarda Munari, Juan Carlo Scirea, Bruno da Cruz Dorneles, Fábio Sprada de Menezes, Filippo Migliorini, Nicola Maffulli, Rodrigo Okubo

**Affiliations:** 1 Physical Therapy Graduate Program, University of the State of Santa Catarina, Florianópolis, SC Brazil; 2 Department of Physiotherapy, University of the State of Santa Catarina, Florianópolis, SC Brazil; 3 Department of Trauma and Reconstructive Surgery, University Hospital of Halle, Martin-Luther University Halle-Wittenberg, Ernst-Grube-Street 40, 06097 Halle (Saale), Germany; 4 Department of Life Sciences, Health, and Health Professions, Link Campus University, Via del Casale di San Pio V, 00165 Rome, Italy; 5 Department of Orthopedics and Trauma Surgery, Academic Hospital of Bolzano (SABES-ASDAA), 39100 Bolzano, Italy; 6 Department of Medicine, Surgery and Dentistry, University of Salerno, 84081 Baronissi, Italy; 7 School of Pharmacy and Bioengineering, Faculty of Medicine, Keele University, Stoke on Trent, ST4 7QB UK; 8 Centre for Sports and Exercise Medicine, Barts and the London School of Medicine and Dentistry, Mile End Hospital, Queen Mary University of London, London, E1 4DG UK

**Keywords:** Postural balance, Athletes, Range of motion, Functional performance, Diagnosis

## Abstract

Dynamic balance is a critical component of athletic performance, especially in soccer, influencing agility, change of direction, and injury prevention. The modified Star Excursion Balance Test (mSEBT) is a reliable and accessible tool to assess dynamic balance in athletes. Poor performance in the mSEBT is correlated with a higher risk of lower limb injuries in young athletes, highlighting the importance of understanding factors influencing its performance. We hypothesised a relationship between ankle dorsiflexion range of motion (ROM) and performance on the mSEBT in healthy young athletes, that could contribute to more precise assessment and intervention in clinical practice and sports. Twenty-six young soccer players participated in this cross-sectional observational study. Ankle dorsiflexion ROM was quantified using the weight bearing lunge test (WBLT), assessed with a digital inclinometer positioned 15 cm from the anterior tibial tuberosity. Following a brief warm-up, athletes performed the mSEBT. Pearson correlation and linear regression analyses were conducted. A robust positive correlation was found between ankle dorsiflexion ROM and anterior reach (*p* = 0.001), with moderate correlations for posteromedial reach (*p* = 0.006) and composite score (*p* = 0.001). A weak correlation was observed for posterolateral reach (*p* = 0.048). Linear regression indicated that anterior reach explained 56% of the variance in dorsiflexion ROM (*R*^2^ = 0.563, *p* < 0.001), allowing a quick estimation of dorsiflexion mobility from a simple field test. Ankle dorsiflexion ROM showed significant correlations with multiple mSEBT directions, mainly influencing anterior reach performance, which accounted for 56% of its variance. These findings highlight the anterior reach of the mSEBT as a practical screening tool to estimate dorsiflexion mobility and support functional performance assessment in young soccer athletes.

## Introduction

Dynamic balance is a critical component of athletic performance, particularly in soccer, where it influences agility, change of direction, and injury prevention [1]. The modified Star Excursion Balance Test (mSEBT) is a reliable and widely used tool for assessing dynamic balance in athletes, serving as a clinical practice assessment measure, a pre-season screening test, and a return-to-sport criterion [2–5]. Poor performance on the mSEBT has been associated with a higher risk of lower limb injuries in young athletes [6], underscoring the importance of identifying factors that influence test performance. Among these, ankle dorsiflexion range of motion (ROM) has been correlated with mSEBT outcomes [7]. This ROM is typically assessed using the weight bearing lunge test (WBLT), a reliable and reproducible method requiring minimal equipment [8]. Moreover, in individuals with functional ankle instability, reduced dorsiflexion ROM has been linked to impaired dynamic balance [7].

Previous studies have reported correlations between ankle dorsiflexion ROM and dynamic balance in populations with chronic ankle instability and other injured cohorts [9, 10]. However, evidence in healthy young athletes is limited and inconsistent [11], highlighting the need for further investigation in this population. Identifying a direct relationship between ankle dorsiflexion ROM and mSEBT performance in healthy young athletes can contribute to more precise assessment and intervention in clinical practice and sports. A better understanding of this relationship can inform the development of targeted interventions to improve balance and reduce injury risk. Therefore, this study investigated the correlation between ankle dorsiflexion ROM and mSEBT performance in young athletes. The novelty of this study lies in the use of a homogeneous cohort of elite U-20 soccer athletes and in providing, for the first time, a regression model linking mSEBT performance to ankle dorsiflexion ROM in this population. We hypothesised that ankle dorsiflexion ROM would be positively correlated with mSEBT performance, particularly in the anterior direction.

## Methods

### Study design

A cross-sectional observational study was conducted with approval from the Local Ethics Committee (CAAE number: 64790922.7.0000.0118). The study investigated the relationship between ankle dorsiflexion ROM and mSEBT performance in 26 male U-20 soccer players playing in a professional Brazilian club. Participants were recruited through convenience sampling.

### Sample

The sample consisted of athletes from the Under-20 (U-20) category of a professional soccer club. Only participants who agreed and signed the consent form participated in the investigation. The inclusion criteria were age between 18 and 20 years, at least 2 years of sports practice, and at least 3 h of weekly training [12]. Exclusion criteria included any trunk or lower limb injury within the last 3 months, a history of surgical procedures, or previous injuries that could affect function and performance in the mSEBT. In the present context, an injury was defined as any musculoskeletal complaint presented by the athlete. This study considered only time-loss injuries, defined as those that resulted in absence from training or competition for more than seven consecutive days. We also noted discomfort or pain during data collection, inability to perform data collection procedures, previous neurological diseases, and vestibular disorders [13–15].

### Sample size

The sample size was estimated using G*Power software (version 3.1.9.7). A medium effect size (Cohen’s d = 0.5) was adopted based on previous studies reporting moderate correlations between ankle dorsiflexion range of motion and dynamic balance performance [9, 16]. With a significance level of 0.05, statistical power of 0.80, and a two-tailed analysis, the minimum required sample size was 21 participants.

### Procedures

Participants were informed verbally and in writing about the study objectives and data collection procedures. After signing the consent form, anthropometric data, limb dominance, and relevant information about sports practice (i.e., years competing at club level under a sports federation license) were collected. Lower limb dominance was defined based on the athlete’s response to the question: “Which leg would you use to kick a ball as hard as possible?” This method is commonly used in sports science; however, it is important to note that the kicking limb does not always correspond to the balance-supporting limb, as the non-dominant leg is often responsible for providing postural stability [17]. The researcher verbally explained and guided the participants through the tests, answering any questions they may have had about the test’s execution. Subjects could stop the tests at any time if they felt pain or discomfort. Assessments of dorsiflexion ROM and the functional test (mSEBT) were performed in this order. All assessments were conducted under standardised conditions, at a similar time of day, barefoot on the same flat, vinyl-coated indoor surface, to ensure consistency. A brief 2-min rest interval was provided between the WBLT and the mSEBT to minimise fatigue. Only the dominant limb was tested to reduce data collection time and participant burden while ensuring methodological consistency across the sample. Previous evidence indicates that limb-to-limb differences are often minimal in healthy athletes, supporting the representativeness of this approach [18, 19].

### Dorsiflexion ROM

**Fig. 1:**
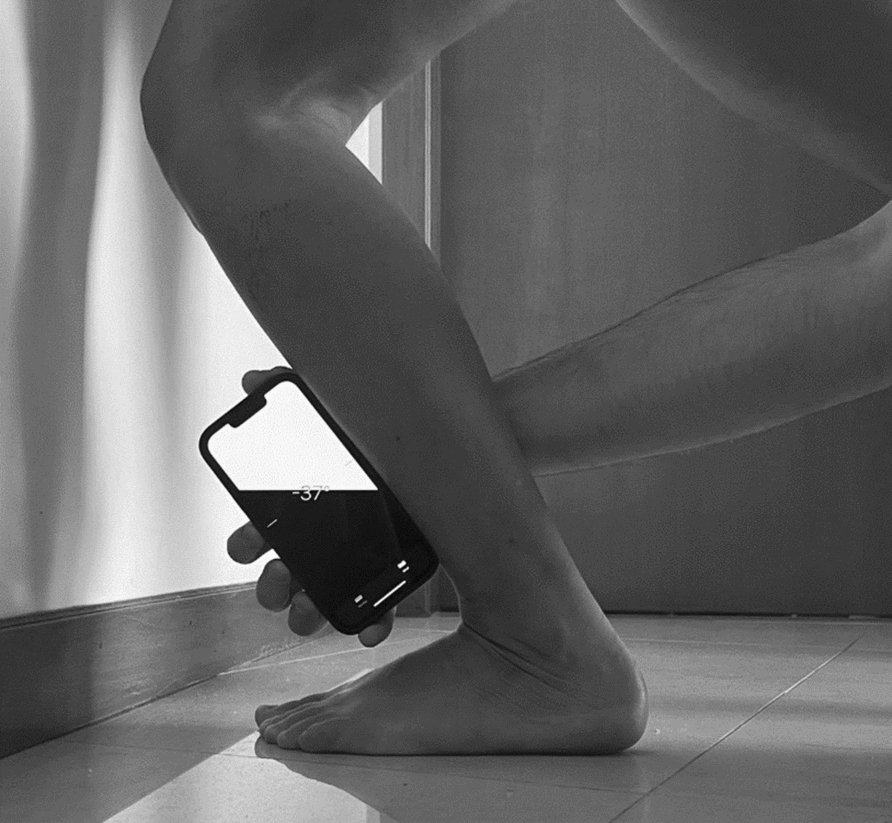
Application of WBLT

Dorsiflexion ROM was measured using the WBLT. The evaluated limb was always the athlete's dominant limb. The athlete was instructed to position their barefoot orthogonally to the wall and attempt to touch their knee to the wall while keeping their feet as far back as possible. They were to ensure that the heel remained on the ground and the chest faced forward towards the wall. Five repetitions were performed for familiarisation. Then, the digital inclinometer of the “Measure App” on an Apple iPhone 13 was positioned 15 cm from the anterior tibial tuberosity and used to measure the maximum dorsiflexion angle (Fig. 1). Before each testing session, the inclinometer was zeroed on a flat surface to verify calibration and measurement accuracy. The validity and reliability of the iPhone level function for the WBLT have been previously demonstrated against a digital inclinometer [20], including an intra-rater ICC of ~ 0.85 in the knee-extended position. The assessment was considered invalid if the heel was lifted off the ground or if there was excessive trunk rotation [8, 21]. The entire test was performed under the supervision of a fully trained healthcare professional, and the same examiner carried out all assessments to ensure consistency and minimise potential inter-rater bias.

### mSEBT

Before performing the mSEBT, the athlete watched a standardised explanatory video with instructions on how to perform the test and was instructed to perform a 5-min warm-up on a cycle ergometer Embreex, model BIKE 364GX, Brusque, Santa Catarina, Brazil. The cadence was 60 rotations per minute (RPM), which could vary between 55 and 65 RPM, generating a power of 1 W per kilogram of body weight. This protocol was chosen, because cycling provides a standardised, low-impact, and reproducible warm-up that activates the lower limb musculature and increases circulation without introducing balance or task-specific exercises that might bias subsequent performance on the mSEBT.

Four repetitions were performed on each side for familiarisation, followed by 1 min of rest [2]. After familiarisation, three valid repetitions were collected for each direction. The test consists of reaching as far as possible in three directions while maintaining a single-leg stance with the contralateral side. For setting up the mSEBT, three measuring tapes were placed on the floor in three different directions: anterior (ANT), posteromedial (PM), and posterolateral (PL) [2]. The anterior tape was positioned 135° apart from the other two, while the posteromedial and posterolateral tapes were 90° apart, forming a “Y” shape [2].

**Fig. 2:**
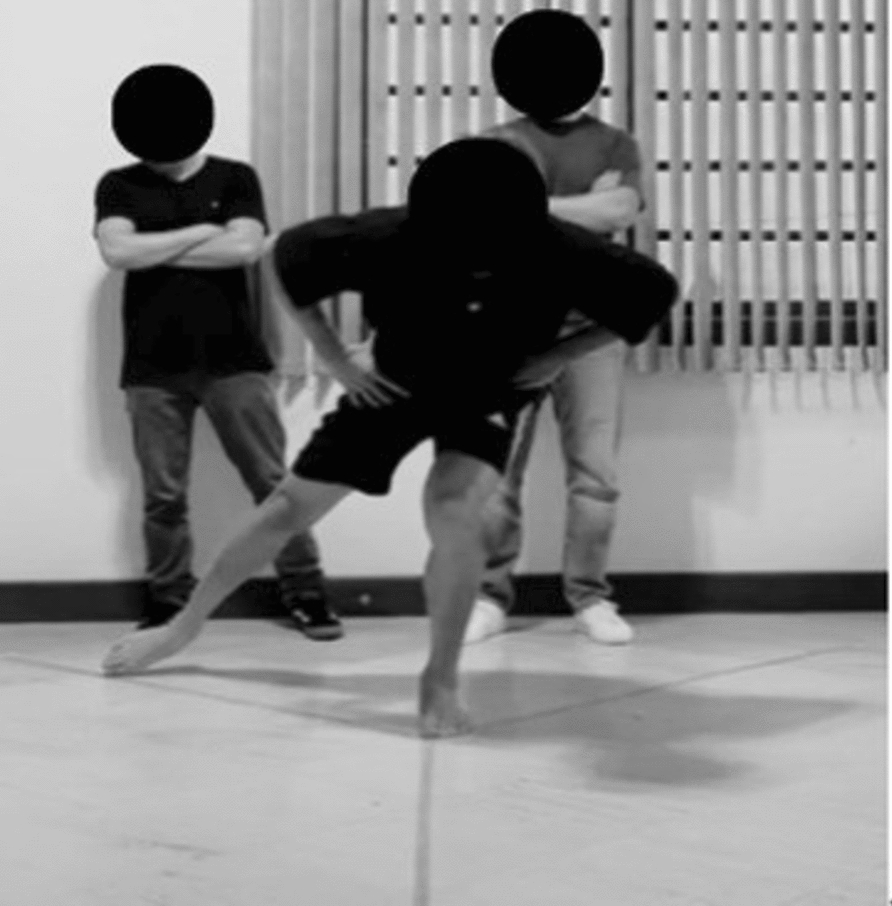
Application of mSEBT

The test was conducted with the athlete positioned with their hands on their hips. The evaluated limb was aligned, so that the most distal portion of the hallux was at the intersection of the three measuring tapes (0°) throughout the procedure (Fig. 2) [2]. For standardisation purposes, all athletes were evaluated barefoot [2]. Two trained and experienced healthcare professionals supervised the entire test.

The average distance reached by the hallux of the swinging limb during the three attempts in each direction of the mSEBT was calculated [2]. These values were then normalised by the athlete’s lower limb length, measured as the distance between the anterior superior iliac spine (ASIS) and the distal tip of the medial malleolus, with the participant in the supine position (distance divided by limb length and multiplied by 100) [2].

### Statistical analysis

Data were analysed using SPSS 20.0 software (SPSS Inc., Chicago, IL), adhering to the CHAMP statement [22]. Descriptive statistics were calculated, and normality was confirmed using the Shapiro–Wilk test. Pearson correlation and linear regression analyses examined the relationship between dorsiflexion ROM and mSEBT performance. All variables met the assumptions of linearity and normal distribution, supporting the application of Pearson correlation and linear regression analyses. The strength of correlation coefficients was classified as negligible (0.00–0.29), low (0.30–0.49), moderate (0.50–0.69), high (0.70–0.89), and very high (0.90–1.00). For the linear regression, assumptions were verified by assessing the normality of residuals (using the Shapiro–Wilk test), homoscedasticity (through visual inspection of residuals versus fitted values), and influential outliers (using Cook’s distance). Statistical significance was set at *p* < 0.05. No formal correction for multiple comparisons was applied, as the four correlation tests (three mSEBT directions and the composite score) were planned a priori.

## Results

**Fig. 3:**
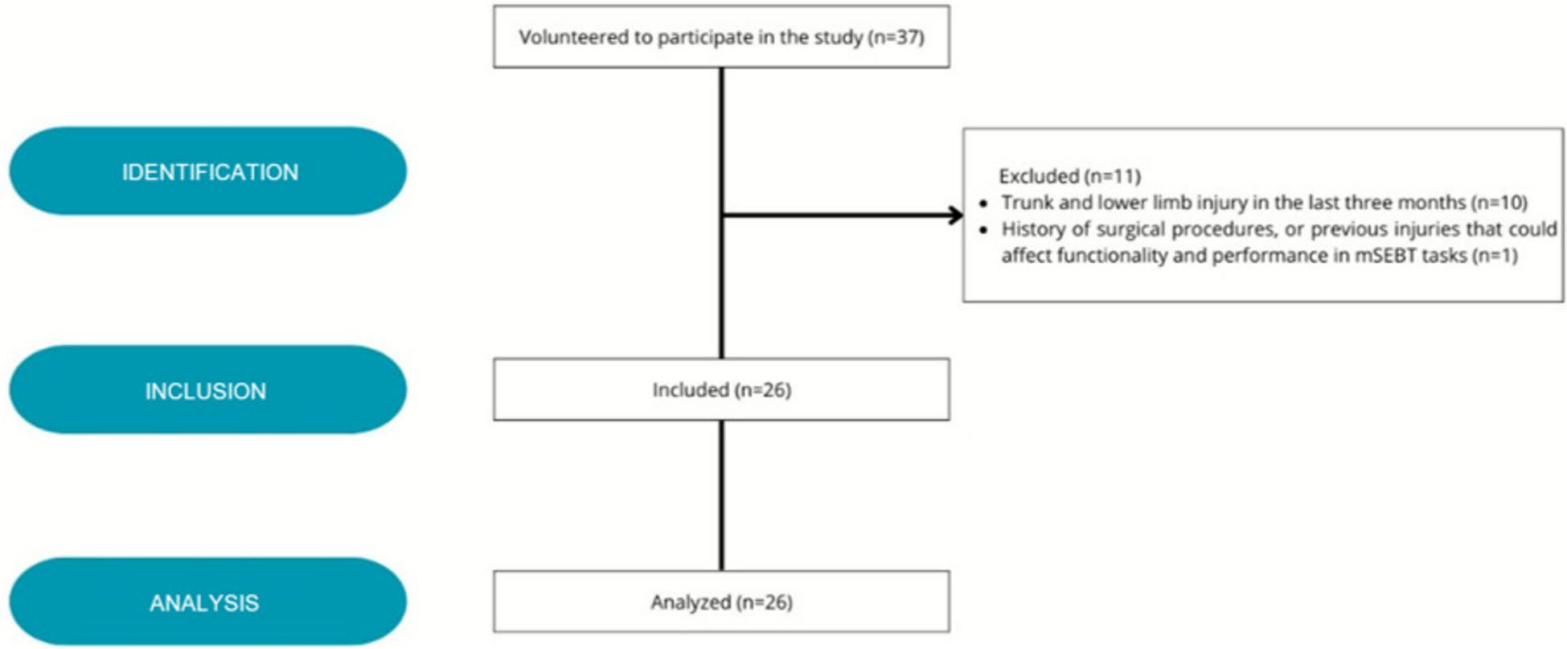
Strobe subject flow diagram indicating inclusion and exclusion criteria

Thirty-seven athletes volunteered to participate in the study, of which 11 did not meet the inclusion criteria (Fig. 3). Twenty-six male soccer players with an average age of 18.7±0.8 years and an average height of 180.2±6.7 cm were included in the analysis. The characteristics of the study cohort are presented in Table 1, and performance on the mSEBT components and dorsiflexion ROM are presented in Table 2.

**Table 1:** Participant demographics and training characteristics

Variable	Mean ± SD	[Min–Max]
Age (years)	18.7 ± 0.8	[18.0–20.0]
Weight (kg)	74.3 ± 6.6	[62.0–86.0]
Height (cm)	180.2 ± 6.7	[169.0–194.0]
Years of federated practice (years)	6.3 ± 3.2	[2.0–12.0]

*SD* = standard deviation, *Min* = minimum, *Max* = maximum, Kg = Kilogram, *cm* = centimetre

**Table 2:** Primary outcome measures (mSEBT scores, dorsiflexion ROM)

Variable	Mean ± SD	[Min–Max]
ANT (%)	61.3 ± 7.6	[44.2–83.5]
PL (%)	99.1 ± 7.2	[81.0–120.0]
PM (%)	97.9 ± 7.8	[81.1–117.6]
Composite score (%)	85.5 ± 5.5	[69.0–95.0]
Dorsiflexion (degrees)	41.3 ± 8.1	[17.0–52.0]

*SD* = standard deviation, *Min* = minimum, *Max* = maximum, % = percentage, *ANT* = Anterior reach, *PL* = Posterolateral reach, *PM* = Posteromedial reach

**Fig. 4:**
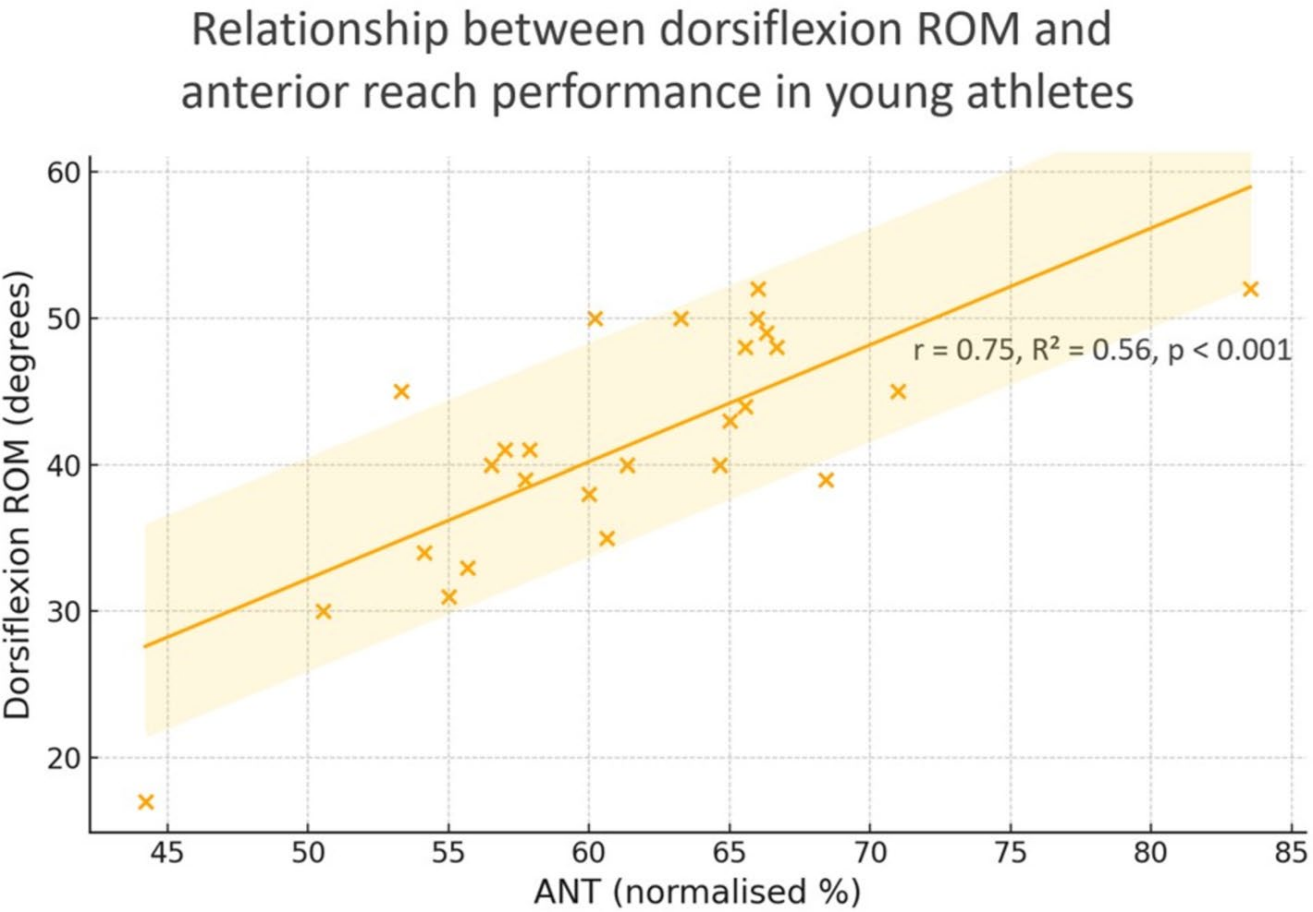
Relationship between ankle dorsiflexion range of motion (ROM, degrees) measured by the weight-bearing lunge test and the anterior reach component of the modified Star Excursion Balance Test (mSEBT, normalised %). The solid line represents the fitted linear regression with the shaded 95% confidence band. *r* = 0.75, *R*^2^ = 0.563, *p* < 0.001

Pearson correlation analyses showed significant correlations between dorsiflexion ROM and mSEBT performance, with effect sizes ranging from low to high: ANT (*r* = 0.751, 95% CI [0.512, 0.882], *p* < 0.001), PL (*r* = 0.391, 95% CI [0.005, 0.676], *p* = 0.048; weak and borderline), PM (*r *= 0.526, 95% CI [0.174, 0.759], *p* = 0.006), and composite score (*r *= 0.656, 95% CI [0.361, 0.832], *p* < 0.001) (Table 3). The relationship between anterior reach and dorsiflexion ROM is illustrated in Fig. 4.

**Table 3:** Correlation between mSEBT components and ankle dorsiflexion range of motion was measured using the WBLT

		ANT	PL	PM	Score
WBLT	Pearson r	0.751	0.391	0.526	0.656
	95% CI	[0.512–0.882]	[0.005–0.676]	[0.174–0.759]	[0.361–0.832]
	*p* value	< 0.001	0.048	0.006	< 0.001
	Strength	High	Low	Moderate	High

*ANT* = Anterior reach, *PL* = Posterolateral reach, *PM* = Posteromedial reach, *WBLT* = Weight-bearing lunge test

Linear regression indicated that ANT explained approximately 56% of the variance in dorsiflexion ROM (*R*^2^ = 0.563, *p* < 0.001).

The resulting Equation (95% CI for slope: 0.502–1.094) was:

$${\mathbf{y}} \, = \, - {\mathbf{7}}.{\mathbf{665}} \, + \, {\mathbf{0}}.{\mathbf{798x}}$$

Y = ankle dorsiflexion ROM in degrees.

x = normalised anterior reach.

All regression assumptions were satisfied (normality: Shapiro–Wilk *p* = 0.919; homoscedasticity: Breusch–Pagan *p* = 0.402), and no highly influential outliers were detected (Cook’s D < 1). A sensitivity analysis excluding three observations with Cook’s D > 4/n yielded similar results (*R*^2^ = 0.515; slope = 0.884). All regression assumptions were met, and no influential outliers were identified, confirming the robustness of the model.

## Discussion

This study is the first to investigate the relationship between mSEBT performance and ankle dorsiflexion range of motion (ROM) in a homogeneous cohort of healthy young soccer athletes providing a regression model that quantitatively links these variables. The novelty of our findings lies in demonstrating that dorsiflexion ROM accounted for a substantial proportion of the variance in the anterior reach component, and in providing a regression formula that allows clinicians to approximate dorsiflexion ROM using a simple, widely applied functional balance test. This integration of joint mobility assessment and functional performance into a single screening strategy offers practical applications for sports performance monitoring and return-to-sport evaluations.

Our results revealed a statistically significant positive correlation between a greater angle of ankle dorsiflexion and better performance in various directions of the mSEBT and its composite score. Ankle ROM plays a crucial role in the ability of young soccer players to achieve better performance in balance demands, potentially reflecting improved stability and neuromuscular control. Including joint ROM assessments in the screening and monitoring routines of young athletes may facilitate more targeted interventions to enhance sports performance and prevent injuries.

Underlying neuromuscular and kinematic mechanisms may partly explain the correlation between dorsiflexion ROM and anterior reach performance. Limited dorsiflexion alters lower limb kinematics and muscle activation patterns during squatting, leading to compensatory strategies that challenge neuromuscular control [23]. Similarly, reduced dorsiflexion has been associated with altered hip and knee kinematics during landing and step-down tasks, which may compromise dynamic stability and postural alignment [24, 25]. These findings suggest that greater dorsiflexion facilitates more efficient biomechanical positioning of the lower limb, reducing compensatory muscle activation and supporting improved dynamic balance during the mSEBT. Moreover, the more decisive influence of dorsiflexion ROM on the anterior reach compared to the posteromedial and posterolateral directions can be explained biomechanically. The anterior reach requires the tibia to translate forward over the fixed foot, directly demanding greater dorsiflexion at the talocrural joint. In contrast, the posteromedial and posterolateral reaches rely more on multiplanar strategies involving hip mobility, trunk leaning, and neuromuscular coordination to maintain stability, which reduces the relative contribution of ankle dorsiflexion. This distinction clarifies why dorsiflexion ROM was a stronger determinant of anterior reach performance in this study, a finding consistent with previous reports showing that anterior reach is particularly sensitive to dorsiflexion restrictions [9, 16].

Our findings are consistent with previous studies which identified similar correlations. For instance, other authors found that 16% of the variance in the mSEBT composite score could be attributed to dorsiflexion ROM, reinforcing the importance of this ROM as a crucial component in evaluating stability and balance [26]. However, other researchers who indicated that dorsiflexion ROM was only correlated with ANT reach and the composite score [15]. In contrast, this study revealed positive correlations across all components, underscoring the broader relevance of dorsiflexion ROM in mSEBT performance. In addition, our findings align with those of Almansoof et al. (2023) [10], who observed a positive relationship between dorsiflexion and hop-test performance in recreational athletes, suggesting a similar functional linkage.

The importance of dorsiflexion ROM in dynamic balance has also been described in individuals with chronic ankle instability. Balance and performance on the mSEBT on the affected side are significantly associated with dorsiflexion ROM, highlighting the impact of this ROM on dynamic balance in clinical populations [16, 27].

From a clinical and sports perspective, the present findings suggest that the anterior reach of the mSEBT can serve as a time-efficient screening tool to approximate dorsiflexion ROM. This approach may allow clinicians and sports practitioners to quickly identify athletes who require a more detailed ankle assessment, improving evaluation routines and guiding targeted interventions. Enhancing dorsiflexion ROM has the potential to support dynamic balance, reduce injury risk, and improve overall athletic performance. Integrating this strategy into return-to-sport protocols may also contribute to safer and more efficient reintegration into training and competition. In practical terms, the anterior reach component of the mSEBT may complement other functional assessment tools, such as the functional movement screen or hop tests, providing clinicians with a quick and integrated approach to identify mobility and stability deficits in athletes.

Clinicians should prioritise interventions to improve ankle dorsiflexion ROM to enhance athlete performance and expedite return-to-sport timelines. Incorporating targeted stretching exercises, mobilisation techniques, and neuromuscular re-education into rehabilitation programs can address limitations in ankle mobility. Regular assessment of ankle dorsiflexion ROM, in conjunction with the mSEBT, can inform treatment planning and monitor progress effectively. By optimising ankle mobility, clinicians can contribute to improved balance, reduced injury risk, and enhanced athletic performance. Recent interventional evidence further supports this approach, showing that corrective exercise programs targeting dorsiflexion can improve balance and proprioception [28].

Finally, the cross-sectional design of the present investigation precludes establishing a causal relationship between ankle dorsiflexion ROM and mSEBT performance. Consequently, longitudinal studies are warranted to elucidate the temporal relationship between these variables. The exclusive focus on young male soccer players limits the generalisability of the findings to other populations, particularly female athletes or individuals engaged in sports other than soccer. In addition, testing was restricted to the dominant limb, which may not capture potential asymmetries between sides. The relatively small sample size also limits the statistical power and external validity of the results. Future research should aim to validate this regression model in female athletes and participants from various sports, ideally employing longitudinal designs to determine whether dorsiflexion ROM predicts changes in dynamic balance or injury risk over time.

## Conclusion

This study demonstrated a significant relationship between ankle dorsiflexion range of motion and performance on the modified Star Excursion Balance Test in young male soccer athletes. The anterior reach component of the mSEBT was mainly influenced by ankle dorsiflexion, accounting for approximately 56% of its variance. The derived regression formula (y = − 7.665 + 0.798x) should be interpreted as a potential screening tool requiring further validation, and not as a diagnostic criterion. Importantly, given the cross-sectional design, no causal inferences can be made. In practice, incorporating the anterior reach component of the mSEBT into athlete screening routines may provide a quick and low-cost strategy to identify restricted ankle mobility and guide targeted interventions.

Future research should investigate the generalisability of these findings to different populations, including female athletes and individuals with varying sports backgrounds, and employ longitudinal designs to explore potential causal relationships between ankle dorsiflexion and injury prevention.

## Data Availability

Data supporting the findings are available from the corresponding author upon reasonable request.
